# Associations Between Different Cortisol Measures and Adiposity in Children: A Systematic Review and Meta-Analysis

**DOI:** 10.3389/fnut.2022.879256

**Published:** 2022-06-23

**Authors:** Lu Ma, Xi Liu, Na Yan, Yiqun Gan, Yue Wu, Ying Li, Meng Chu, Dorothy T. Chiu, Le Ma

**Affiliations:** ^1^School of Public Health, Global Health Institute, Xi’an Jiaotong University Health Science Center, Xi’an, China; ^2^School of Public Health, Xi’an Jiaotong University Health Science Center, Xi’an, China; ^3^The First Affiliated Hospital of Xi’an Jiaotong University, Xi’an, China; ^4^Beijing Key Laboratory of Behavior and Mental Health, School of Psychological and Cognitive Sciences, Peking University, Beijing, China; ^5^Department of Cardiology, The First Affiliated Hospital of Xi’an Jiaotong University, Xi’an, China; ^6^Key Laboratory of Environment and Genes Related to Diseases, Ministry of Education, Xi’an, China; ^7^Key Laboratory of Molecular Cardiology, Xi’an, China; ^8^Department of Cardiology, The Second Affiliated Hospital of Xi’an Jiaotong University, Xi’an, China; ^9^Community Health Sciences Division, School of Public Health, University of California, Berkeley, Berkeley, CA, United States

**Keywords:** hair cortisol concentration, salivary cortisol, serum cortisol, urinary cortisol, obesity, children

## Abstract

This study examined associations between hair, salivary, serum, and urinary cortisol concentration with adiposity-related indicators in children, and explored their potential effects modification by age, sex, cortisol measurement method, and country developmental context. We systematically searched PubMed, Web of Science, and Embase for studies examining at least one of the four aforementioned cortisol with objectively measured adiposity-related outcomes in children. Meta-analyses of cross-sectional studies revealed that hair cortisol concentration was associated with fat mass index (FMI)-standard deviation score (SDS)/FMI z-score (pooled-β = 0.04, 95% CI: 0.01, 0.08) and BMI/BMI z-score (pooled-β = 0.15, 95% CI: 0.06, 0.25), and these associations were significant among children aged ≤ 12 years (pooled-β = 0.15, 95% CI: 0.05, 0.26) and >12 years (pooled*-*β = 0.13, 95% CI: 0.04, 0.22), children from developed countries (pooled β = 0.12, 95% CI: 0.03, 0.21) and developing countries (pooled-β = 0.193, 95% CI: 0.188, 0.198), and in studies extracting cortisol *via* LC-MS/MS (pooled-β = 0.18, 95% CI: 0.06, 0.29) but not ELISA (pooled-β = 0.08, 95% CI: −0.06, 0.22). Meta-analyses of both cohort and cross-sectional studies revealed non-significant associations of morning salivary cortisol concentration and total daily cortisol output with BMI/BMI z-score. Serum cortisol concentration was not associated with BMI or waist circumference. Meta-analysis of urinary cortisol concentration and adiposity was hindered by insufficient data. These findings further corroborate understanding of chronic stress’ physiological contribution to increased pediatric obesity risk.

**Systematic Review Registration:**

[https://www.crd.york.ac.uk/prospero/#recordDetails], identifier [CRD42020215111].

## Introduction

Childhood obesity persists as a global public health crisis (1–4). Recent research has identified stress as an important risk factor for childhood adiposity (5–8). Stress is a negative emotional experience accompanied by predictable biochemical, physiological, cognitive, and behavioral changes directed toward altering the stressful event or accommodating to its effects (7); such changes may further serve to increase childhood obesity risk (7). Measurement of stress is inherently complex and requires consideration of multiple dimensions, including the social, psychological, and physiological (9). Given the inherent limitations of using subjective, self-reported measures for stress, considerable literature has established the use of physiological biomarkers for the objective assessment of stress for research. However, associations between physiological measures of stress and adiposity-related indicators in children are inconsistent, preventing a unified understanding of the stress processes in childhood obesity and subsequent design of related interventions.

The hypothalamic-pituitary-adrenal (HPA) axis is the most widely studied physiological stress system. When an individual perceives stress, a physiological cascade occurs in the HPA axis, and its main downstream hormone “cortisol” has been viewed as the “gold standard” biomarker with which to assess stress (6, 10). Alterations in HPA axis may be reflected in changes in the level and diurnal trajectory of cortisol secretion (11). Cortisol can facilitate obesity by stimulating unhealthy eating behaviors and promoting fat deposition (7). Moreover, visceral adipose tissue itself is rich in 11β-hydroxysteroid dehydrogenase type I, which converts inactive cortisone to cortisol (12). Therefore, a potential bidirectional relationship between cortisol and adiposity outcomes may exist. However, in this study, our primary focus will be placed on examinations of cortisol on adiposity outcomes in children.

It is possible for laboratories to utilize blood, urine, saliva, and hair to measure cortisol (13). For many years, cortisol was obtained primarily from serum or urine, but more recent approaches have sampled saliva and hair for less invasive monitoring of HPA functioning, and each measure reflects bodily cortisol levels. Serum cortisol concentration measures the total cortisol (14). Salivary cortisol concentration is usually used to assess the circadian rhythm of cortisol (e.g., cortisol awakening response) and the secretion of cortisol under stress-induced conditions (e.g., the total output of cortisol) (15). Urine samples will generally capture HPA activity over a period of only 24 h or less. In contrast, hair cortisol concentration (HCC) have increasingly been used to assess the long-term presence and/or accumulation of cortisol in children (16, 17).

More research is needed to evaluate and understand the associations between different cortisol measures for stress with adiposity-related outcomes in children. However, the literature on such associations is very limited (18–20). To date, only one systematic review (of *n* = 26 studies) has provided the evidence on associations between HCC and obesity in children, finding a modest positive correlation between HCC and anthropometric measures including body mass index (BMI), BMI z-score, waist circumference (WC), and body fat (21). However, the meta-analyses of reviewed studies did not exclude those relying on self-reported weight status and did not distinguish between cross-sectional and longitudinal studies. Moreover, studies have suggested that individual (e.g., age and sex) and environmental contextual factors (e.g., country development status) may modify associations between cortisol and adiposity outcomes in children (22). For example, a study found that association between cortisol and increased BMI were stronger in early adolescence than in late adolescence (23). Another study showed that altered cortisol balance modified the net lipogenetic/lipolytic in various adipose tissue depots in a sex-dependent manner in the periphery, therefore contributing to the differential associations between cortisol and adiposity outcomes (24). Furthermore, lower socio-economic status of a country was a predictor of higher cortisol levels and obesity risk (25, 26). These findings indicate that these background factors may modify the associations between cortisol and adiposity-related outcomes in children. Interestingly, no studies have heretofore examined how different cortisol measures may vary in their associations with obesity by different sociodemographic or socio-economic factors in children.

Therefore, this systematic review and meta-analysis aimed to examine associations of different cortisol measures – hair, saliva, serum, and urine – with various adiposity-related outcomes in children, and to further explore the potential modification of these associations by external contextual factors including child age, sex, cortisol measurement method, and country developmental context. These findings will synthesize the body of evidence surrounding associations between different cortisol measures and pediatric obesity, and advance the understanding of child stress biomarker research.

## Methods

This study was developed and reported according to the Preferred Reporting Items for Systematic Reviews and Meta-Analyses (PRISMA) and other recommended practice standards (e.g., Johnson and Hennessy, 2019).

### Literature Search

A systematic search was performed in three electronic bibliographic databases-PubMed, Web of Science, and Embase-for relevant studies published from inception to October 2021. We developed a search strategy for databases based on keywords of seminal articles we had previously identified. Search strategies included all combinations of terms related to cortisol, anthropometric measures, and children (Supplementary Table 1).

Hand searching of references was conducted to uncover any potentially overlooked studies. Articles identified from the reference lists were further screened and evaluated using the same study criteria. Reference searching was repeated on all newly identified articles until no additionally relevant articles were found.

### Study Selection

Studies that met all of the following criteria were included: (1) was cross-sectional, case-control, or longitudinal; (2) studied children under 18 years old without mental disorders or any diagnosed chronic conditions (e.g., hypertension, cardiovascular disease); (3) examined naturally occurring cortisol, assayed from either urine, saliva, hair or blood, as exposure variables; (4) analyzed objectively measured adiposity-related outcomes; (5) reported statistical associations between cortisol and adiposity-related outcomes; (6) were published in English; and (7) were peer-reviewed publications. When multiple articles reported on the same data, the article with the largest sample size and results most relevant to this review was retained. Two authors assessed all identified studies for eligibility independently and disagreements were resolved through discussion.

### Data Extraction and Preparation

A standardized form was developed to collect information from selected studies. Data extracted included that on: (1) the study (e.g., first author, publication year, study design, cortisol measure[s] used, adiposity-related outcome[s] assessed, the country site of study, and the country site’s developmental context [developed vs. developing]), (2) the sample (e.g., participant age, sex, race/ethnicity), and (3) effect sizes. Acceptable adiposity-related outcomes included BMI/BMI z-score/BMI-standard deviation score (BMI-SDS), waist circumference (WC), percentage body fat (PBF), fat mass index (FMI)-SDS/FMI z-score, free fat mass index (FFMI), and waist to height ratio (WtHR), and truncal distribution of fat mass (TDFM). Data were extracted independently by two authors and discrepancies were resolved through discussion.

### Study Quality Assessment

Two authors independently assessed the quality of eligible articles using the U.S. National Heart, Lung, and Blood Institute’s Quality Assessment Tool for Observational Cohort and Cross-Sectional Studies (27). This assessment tool rates studies based on 14 criteria. For each criterion, a score of one was assigned for “yes” and zero otherwise (i.e., “no,” “not applicable,” “not reported,” or “cannot determine”). Overall quality was rated based on the total score of the scale, with 0–3, 4–7, and 7–14 reflecting poor, fair, and good quality, respectively. Discrepancies on study quality ratings were also resolved through discussion (Supplementary Table 2).

### Statistical Analysis

A meta-analysis was performed to estimate the pooled associations between different cortisol measures and adiposity-related outcomes in children. Study heterogeneity was assessed using the I^2^ index and Tau-squared (T^2^). The level of heterogeneity represented by I^2^ was interpreted as modest (I^2^ ≤ 25%), moderate (25% < I^2^ ≤ 50%), substantial (50% < I^2^ ≤ 75%), or considerable (I^2^ > 75%) (28). A random-effects model was applied because of assumed clinical and methodological heterogeneity among the studies (29).

Pre-specified subgroup analyses were conducted to test potential modifying effects of age, sex, country developmental context (i.e., developed vs. developing), and cortisol measurement method [i.e., enzyme-linked immunosorbent assay (ELISA) vs. liquid chromatography tandem-mass spectrometry (LC-MS/MS) vs. chemiluminescence immunoassay (CLIA) vs. electrochemiluminescence immunoassay (ECLIA) vs. Radioimmunoassay (RIA) vs. dissociation-enhanced lanthanide fluorescence immunoassay (DELFIA) vs. a time-resolved fluorescence immunoassay (TRFIA)]. Sensitivity analyses were conducted to investigate the influence of a single study on the overall pool estimation by omitting one study at a time.

Publication bias was assessed by visual inspection for symmetry/asymmetry of contour-enhanced funnel plots and Egger’s tests. All statistical analyses were conducted in Stata 14 with specific meta-analysis commands (i.e., metan and metareg) (College Station, TX, United States). All analyses used two-sided tests and *p* < 0.05 was considered statistically significant.

## Results

### Study Selection

**FIGURE 1:**
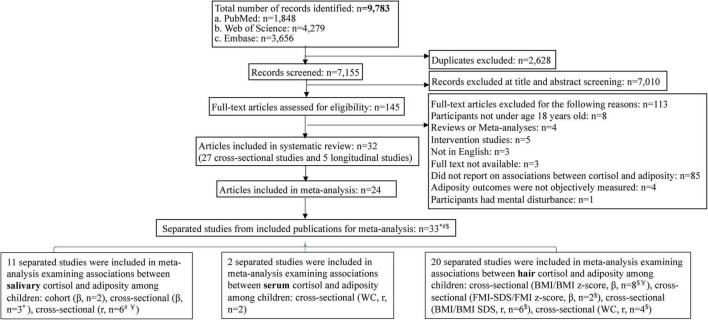
Flowchart of the literature search and study selection procedures. *Included articles were divided into separate studies by different age groups. ^#^Included publications were divided into separate studies by gender. ^$^Included articles were divided into separate studies by different indicators of adiposity. ^Y=^Included articles were divided into separate studies by different measurement of cortisol.

The search identified 8,627 articles of which 38 (31 cross-sectional articles and seven longitudinal articles) were included in this systematic review, with a sample size of 18,667 children. Twenty-four articles were included in the meta-analysis (Figure 1). For testing potential modifying effects, nine (20, 30–37) of the 24 articles were further divided into 18 separate studies given differences in age, sex, indicators of adiposity, and cortisol measurement method, thus, in sum, 33 separated studies were included for meta-analysis. Study characteristics are shown in Table 1.

**TABLE 1:** Summary of main characteristics of 33 studies reporting on associations between hair, salivary, serum, and/or urinary cortisol concentration with adiposity-related outcomes in children.

First author, Publication year; Country and development context	Study design	Sample size (% Girls)	Age (years, Mean ± SD, Range)	Race/Ethnicity	Cortisol measure		Adiposity outcomes		Effect size/Associations between cortisol and adiposity/Covariates
							
					Measure source	Measurement method	Measures	Method of ascertainment	
**Hair cortisol**									
1^ 1^ Vehmeijer et al. (55); Netherlands (Developed)	Cohort	2,042 (52.5%)	5.90 (5.70–8.00)	European and non-European	Hair, 3 cm	LC-MS/MS	BMI-SDS	BMI was calculated based on measured weight and height. BMI-SDS was generated based on Dutch reference growth charts	Increase of BMI SDS (β = 0.06, 95% CI: 0.02, 0.09) per quintile of hair cortisol Covariates included: (1) Child’s: Sex and age, maternal pre-pregnancy BMI, psychological distress during pregnancy (2) Maternal: Educational level and marital status at 6 years, child’s ethnicity, hair color and television watching time
1^2^ Vehmeijer et al. (55); Netherlands (Developed)	Cohort	2,042 (52.5%)	5.90 (5.70–8.00)	European and non-European	Hair, 3 cm	LC-MS/MS	FMI-SDS	FMI was measured by DXA	Increase of FMI-SDS (β = 0.05, 95% CI: 0.02, 0.08) per quintile of hair cortisol Covariates included: (1) Child’s: Sex and age, maternal pre-pregnancy BMI, psychological distress during pregnancy (2) Maternal: Educational level and marital status at 6 years, child’s ethnicity, hair color and television watching time
1^3^ Vehmeijer et al. (55); Netherlands (Developed)	Cohort	2,042 (52.5%)	5.90 (5.70–8.00)	European and non-European	Hair, 3 cm	LC-MS/MS	Overweight vs. Non-overweight	BMI was calculated by measuring weight and height. Weight status was defined based on the International Obesity Task Force cut-offs, the age- and sex- specific cut-off points	Increased risk for overweight or obesity of (OR = 1.18, 95% CI: 1.07, 1.29) per quintile of hair cortisol Covariates included: (1) Child’s: Sex and age, maternal pre-pregnancy BMI, psychological distress during pregnancy (2) Maternal: Educational level and marital status at 6 years, child’s ethnicity, hair color and television watching time
2^1^ Bethancourt et al. (56); Bolivia (Developing)	Cross-sectional	167 (53.2%)	9.70 (6.00–15.00)	Not reported	Hair, 1.5 cm	ELISA	BMI z-score	BMI was calculated based on measured weight and height. BMI z-score was generated based on the WHO reference values and macros	Increase of −0.02 BMI z-score (SE = 0.02, *p* = 0.26) per 20% increase of hair cortisol Covariates included: Maternal: Community and household of residence, age, household adult equivalents, household income, self-reported perceived social status
2^2^ Bethancourt et al. (56); Bolivia (Developing)	Cross-sectional	167 (53.2%)	9.70 (6.00–15.00)	Not reported	Hair, 1.5 cm	ELISA	Body fat percentage	Body fat percentage was measured using a Tanita BF-680W bioelectric impedance scale	Increase of −0.29 percentage of body fat (SE = 0.12, *p* = 0.01) per 20% increase of hair cortisol Covariates included: Maternal: Community and household of residence, age, household adult equivalents, household income, self-reported perceived social status
^#^3^1^ Petimar et al. (57); United States (Developed)	Cohort	491 (NR)	7.80–13.10	White	Hair, ≥3 cm	LC-MS/MS	BMI z-score	BMI was calculated based on measured weight and height. BMI z-score was generated based on US CDC growth charts (2000)	Associations between log hair cortisol concentration (HCC) and BMI z-score: β = 0.00, 95% CI: −0.08, 0.07 Covariates included: Child’s: Age, sex, birthweight-for-sex-and-gestational age *z* score, second-hand smoke exposure, mid-childhood pubertal development score, and early childhood BMI z-score Maternal: enrollment age, maternal education, pre-pregnancy BMI, maternal smoking during pregnancy, household income
^#^3^2^ Petimar et al. (57); United States (Developed)	Cohort	493 (NR)	7.80–13.10	White	Hair, ≥3 cm	LC-MS/MS	Waist circumference	Waist circumference was measured	Associations between log HCC and WC: β = −0.04, 95% CI: −0.83, 0.74 Covariates included: Child’s: Age, sex, birthweight-for-sex-and-gestational age *z* score, second-hand smoke exposure, mid-childhood pubertal development score, and early childhood BMI z-score Maternal: enrollment age, maternal education, pre-pregnancy BMI, maternal smoking during pregnancy, household income
^#^3^3^ Petimar et al. (57); United States (Developed)	Cohort	491 (NR)	7.80–13.10	White	Hair, ≥3 cm	LC-MS/MS	Waist to height ratio (WtHR)	WtHR was calculated based on measured waist and height	Associations between log HCC and WtHR: β = 0.002, 95% CI: −0.003, 0.007 Covariates included: Maternal: enrollment age, education, pre-pregnancy BMI, maternal smoking during pregnancy, household income Child’s: Age, sex, birth weight-for-sex-and-gestational age *z* score, second-hand smoke exposure, mid-childhood pubertal development score, and early childhood waist to height ratio
4 Bryson et al. (58); Australia (Developed)	Cross-sectional	297 (60.6%)	3.10 ± 0.10	Not reported	Hair, 3 cm	ELISA	BMI z-score	BMI was calculated based on measured weight and height. BMI z-score was generated based on Cole’s international criteria (Cole, Bellizzi, 2000)	Associations between HCC and BMI z-score: β = 0.76, 95% CI: 0.51, 1.12, *p* = 0.16 Covariates included: Age, gender, season of assessment, site of hair collection, randomized controlled trial randomization status
5 Baan et al. (59); Netherlands (Developed)	Cross-sectional	298 (45.64%)	12.60–13.20	Not reported	Hair, 3 cm	LC-MS/MS	BMI z-score	BMI was calculated based on measured weight and height. BMI z-score was generated based on the 1997 Dutch nationwide growth study	Associations between log HCC and BMI z-score: β = 0.13, 95% CI: 0.04, 0.22, *p* = 0.01 No covariates were reported
6^1^ Smith et al. (36); Australia (Developed)	Cross-sectional	128 (68.0%)	8.44 ± 0.34	Not reported	Hair, 3 cm	ELISA	Waist circumference	Waist circumference was measured	Correlation between log HCC and WC: *r* = 0.015 No covariates were reported
6^2^ Smith et al. (36); Australia (Developed)	Cross-sectional	128 (68.0%)	8.44 ± 0.34	Not reported	Hair, 3 cm	ELISA	BMI	BMI was calculated based on measured weight and height	Correlation between log HCC and BMI: *r* = −0.047 No covariates were reported
7 Evans et al. (19); Sweden (Developed)	Cross-sectional	92 (NR)	10	Dutch	Hair, 3 cm	LC-MS/MS	BMI	BMI was calculated based on measured weight and height	Correlation between HCC and BMI: *r* = 0.01 No covariates were reported
#8 Distel et al. (18); United States (Developed)	Cohort	52 (61%)	6–10	Mexican	Hair, NR	ELISA	BMI	BMI was calculated based on measured weight and height	Associations between HCC and BMI: β = 4.62, 95% CI: 1.41, 7.83, *p* < 0.01 Covariates included: Age and food insecurity
9 Sun et al. (60); China (Developing)	Cross-sectional	1,000 (57.9%)	8.97 ± 0.86	Han Chinese	Hair, ≥10 mg	ELISA	BMI	BMI was calculated based on measured weight and height	Associations between HCC and BMI: β = 0.17, 95% CI: 0.05, 0.29 Covariates included: PRS polygenic risk score
10 Lu et al. (34); China (Developing)	Cross-sectional	85 (45.9%)	11.40 ± 0.30	Chinese, not otherwise specified	Hair, 3 cm	LC-MS/MS	BMI z-score	BMI was calculated based on measured weight and height. BMI z-score was defined as the number of standard deviation units from the mean or reference value	Associations between log HCC and BMI: β = 0.193, 95% CI: 0.19, 0.20, *p* = 0.004 No covariates were reported
11 Papafotiou et al. (35); Greece (Developed)	Cross-sectional	50 (100%)	7.60 ± 1.30	Greek	Hair, 3 cm	LC-MS/MS	BMI z-score	BMI was calculated based on measured height and weight. BMI z-score was generated based on Cole’ s international criteria (Cole, Bellizzi, 2000)	Correlations between HCC and BMI z-score: *r* = 0.327, *p* = 0.025 No covariates were reported
12^1^ Gerber et al. (32); Switzerland (Developed)	Cross-sectional	318 (53.1%)	7.26 ± 3.51	Not reported	Hair, 3 cm	CLIA	BMI	BMI was calculated based on measured weight and height	Correlations between HCC and BMI: *r* = 0.16, *p* < 0.01, *r* = 0.13 for boys, *r* = 0.16 for girls (*p* < 0.05) No covariates were reported
12^2^ Gerber et al. (32); Switzerland (Developed)	Cross-sectional	318 (53.1%)	7.26 ± 3.51	Not reported	Hair, 3 cm	CLIA	Body fat percentage	Percentage body fat was calculated based on measured skinfold	Correlations between HCC and PBF: *r* = 0.14, *p* < 0.01, *r* = 0.12 for boys, *r* = 0.16 for girls (*p* < 0.05) No covariates were reported
12^3^ Gerber et al. (32); Switzerland (Developed)	Cross-sectional	318 (53.1%)	7.26 ± 3.51	Not reported	Hair, 3 cm	CLIA	Waist circumference	Waist circumference was measured	Correlations between HCC and WC: *r* = 0.14, *p* < 0.01, *r* = 0.18, *p* < 0.05 for boys, *r* = 0.11 for girls No covariates were reported
13 Rippe et al. (25); Netherlands (Developed)	Cross-sectional	2,484 (51.7%)	6.20 ± 0.70	Danish-Caucasian Western and other European	Hair, 3 cm	LC–MS/MS	BMI	BMI was calculated based on measured weight and height	Associations between log HCC and BMI: (95% CI) = 0.025 (0.02, 0.03; *p* = 0.001) No covariates were reported
14 Olstad et al. (61); Australia (Developed)	Cross-sectional	30 (43.3%)	14.30 ± 3.90	Not reported	Hair, 3 cm	ELISA	BMI z-score	BMI was calculated based on measured weight and height. BMI z-score was generated based on the CDC growth charts of U.S (2000)	Associations between HCC and BMI z-score: β = 0.20, 95% CI: −0.85, 1.25, *p* = 0.694 Covariates included: (1) Childs’s: Age (2) Maternal: BMI, education
15^1^ Noppe et al. (20); Netherlands (Developed)	Cross-sectional	2,953 (51.9%)	6.20 ± 0.60	European and non-European, not otherwise specified	Hair, 3 cm	LC–MS/MS	BMI	BMI was calculated based on measured weight and height	Associations between HCC and BMI: β = 0.19, 95% CI: 0.12, 0.26 Covariates included: Child’s: Age, sex, ethnicity, and topical glucocorticoid use
15^2^ Noppe et al. (20); Netherlands (Developed)	Cross-sectional	2,953 (51.9%)	6.20 ± 0.60	European and non-European, not otherwise specified	Hair, 3 cm	LC–MS/MS	FMI-SDS	Fat mass index was measured by DXA	Associations between HCC and FMI: β = 0.05, 95% CI: 0.01, 0.09 Covariates included: Child’s: Age, sex, ethnicity, and topical glucocorticoid use
16 Murray et al. (62); Australia (Developed)	Cross-sectional	95 (52.6%)	9.50 ± 0.34	Not reported	Hair, 3 cm	ELISA	BMI	BMI was calculated based on measured weight and height	Correlations between HCC and BMI: *r* = −0.26 No covariates were reported
17^1^ Larsen et al. (33); Denmark (Developed)	Cross-sectional	317 (NR)	5 (4–7)	Danish, not otherwise specified	Hair, 1–2 cm	ELISA	BMI z-score	BMI was calculated based on measured weight and height. BMI z-score was generated using the Lambda-Mu-Sigma method	Associations between HCC and BMI z-score: β = 0.01, 95% CI: −0.04, 0.07, *p* = 0.70 Covariates included: Child’s: Intervention status, gender, physical activity, maternal education level, and age
17^2^ Larsen et al. (33); Denmark (Developed)	Cross-sectional	280 (NR)	5 (4–7)	Danish, not otherwise specified	Hair, 1–2 cm	ELISA	FMI z-score	FMI was measured by BIA-method, and calculated based on an equation described by Goran et al. (1996) in young Children	Associations between HCC and FMI z-score: β = 0.03, 95% CI: −0.03, 0.08, *p* = 0.32 Covariates included: Child’s: Intervention status, gender, physical activity, maternal education, and age
17^3$^ Larsen et al. (33); Denmark (Developed)	Cross-sectional	280 (NR)	5 (4–7)	Danish, not otherwise specified	Hair, 1–2 cm	ELISA	FFMI z-score	FFMI was calculated by subtracting FFM from body weight. FMI was measured by BIA-method	Associations between HCC and FMI z-score: β = −0.01, 95% CI: −0.07, 0.05, *p* = 0.69 Covariates included: Child’s: Intervention status, gender, physical activity, maternal education, and age
17^4$^ Larsen et al. (33); Denmark (Developed)	Cross-sectional	309 (NR)	5 (4–7)	Danish, not otherwise specified	Hair, 1–2 cm	ELISA	Waist circumference	BMI was calculated based on measured weight and height. BMI z-score was generated using the Lambda-Mu-Sigma method	Associations between HCC and WC: β = 0.10, 95% CI: −0.09, 0.30, *p* = 0.30 Covariates included: Child’s: Intervention status, gender, physical activity, maternal education, and age
17^5$^ Larsen et al. (33); Denmark (Developed)	Cross-sectional	308 (NR)	5 (4–7)	Danish, not otherwise specified	Hair, 1–2 cm	ELISA	WtHR	WtHR was calculated based on measured waist circumference and height	Associations between HCC and WtHR: β = −0.001, 95% CI: −0.003, 0.002, *p* = 0.52 Covariates included: Child’s: Intervention status, gender, physical activity, maternal education, and age
18^1^ Veldhorst et al. (37); Netherlands (Developed)	Cross-sectional	40 (75%)	8–12	Caucasian, no-Caucasian	Hair, 1 cm	ELISA	BMI-SDS	BMI was calculated based on measured weight and height. BMI SDS was generated based on the 2010 Dutch nationwide growth study	Correlations between log HCC and BMI-SDS: *r* = 0.407, *p* < 0.01 No covariates were reported
18^2^ Veldhorst et al. (37); Netherlands (Developed)	Cross-sectional	40 (75%)	8–12	Caucasian, no-Caucasian	Hair, 1 cm	ELISA	Waist circumference	Waist circumference was measured	Correlations between log HCC and WC: *r* = 0.43, *p* < 0.01 No covariates were reported
19^1^ Noppe et al. (63); Netherlands (Developed)	Cross-sectional	128 (50.8%)	8.40 (4.25–14.13)	Not reported	Hair, 3 cm	ELISA	Waist circumference	Waist circumference was measured	Correlations between log HCC and WC: *r* = 0.19, *p* = 0.04 No covariates were reported
19^2^ Noppe et al. (63); Netherlands (Developed)	Cross-sectional	128 (50.8%)	8.40 (4.25–14.13)	Not reported	Hair, 3 cm	ELISA	WtHR	WtHR was calculated based on measured waist circumference and height	Correlations between log HCC and WtHR: *r* = 0.19, *p* = 0.04 No covariates were reported
19^3^* Noppe et al. (63); Netherlands (Developed)	Cross-sectional	128 (50.8%)	8.40 (4.25–14.13)	Caucasian	Hair, 3 cm	ELISA	BMI	BMI was calculated based on measured weight and height	NR
**Salivary cortisol**									
1^1^ Pruszkowska-Przybylska et al. (64); Poland (Developing)	Cross-sectional	73 (100%)	8.92 (7–11)	Not reported	Saliva (8 a.m.–2 p.m.)	ELISA	Body fat percentage	Body fat percentage was measured using the BIA-method	Association between salivary cortisol and FM% was β = −0.089, SE = 0.12, *p* = 0.462 Covariates included: Child’s: Vitamin D concentration Maternal: Education, 2D:4D digit ratio, socio-economic status
1^2^ Pruszkowska-Przybylska et al. (64); Poland (Developing)	Cross-sectional	73 (100%)	8.92 (7–11)	Not reported	Saliva (8 a.m.–2 p.m.)	ELISA	BMI z-score	BMI was calculated based on measured weight and height. The calculated method of BMI z-score was not reported	Association between salivary cortisol and BMI z-score was β = −0.027, SE = 0.117, *p* = 0.818 Covariates included: Child’s: Vitamin D concentration Maternal: Education, 2D:4D digit ratio, socio-economic status
1^3^ Pruszkowska-Przybylska et al. (64); Poland (Developing)	Cross-sectional	60 (0%)	8.92 (7–11)	Not reported	Saliva (8 a.m.–2 p.m.)	ELISA	Body fat percentage	Body fat percentage was measured using the BIA-method	Association between salivary cortisol and FM% was β = −0.091, SE = 0.137, *p* = 0.511 Covariates included: Child’s: Vitamin D concentration Maternal: Education, 2D:4D digit ratio, socio-economic status
1^4^ Pruszkowska-Przybylska et al. (64); Poland (Developing)	Cross-sectional	60 (0%)	8.92 (7–11)	Not reported	Saliva (8 a.m.–2 p.m.)	ELISA	BMI z-score	BMI was calculated based on measured weight and height. The calculated method of BMI z-score was not reported	Association between salivary cortisol and BMI z-score was β = −0.148, SE = 0.134, *p* = 0.273 Covariates included: Child’s: Vitamin Concentration Maternal: Education, 2D:4D digit ratio, socio-economic status
2^1^ Dai et al. (65); United States (Developed)	Cross-sectional	689 (53.0%)	9.20 (SD = 0.41)	Caucasian, not otherwise specified	Saliva (waking, 30 mins post-waking)	ELISA	Body composition	Body composition was indexed by BMI and waist-to-hip ratio. BMI was calculated based on measured weight and height	Salivary cortisol was associated with body composition: β = −0.20, SE = 0.05, *p* < 0.01 Covariates included: Child’s: Sex, age, race, socioeconomic, and medication use
2^2^ Dai et al. (65); United States (Developed)	Longitudinal	647 (55.0%)	10.53 (SD = 0.52)	Caucasian, not otherwise specified	Saliva (waking, 30 mins post-waking)	ELISA	Body composition	Body composition was indexed by BMI and waist-to-hip ratio. BMI was calculated based on measured weight and height	Salivary cortisol at baseline was associated with body composition at follow-up: β = 0.00, SE = 0.02, *p* > 0.05 Covariates included: Child’s: Sex, age, race, socioeconomic, and medication use
3 Pruszkowska-Przybylska et al. (66); Poland (Developing)	Cross-sectional	132 (56.8%)	6–13	Not reported	Saliva (8 a.m.–2 p.m.)	ELISA	Fat mass percentage	Fat mass was measured by BIA-method	Salivary cortisol was associated with fat mass percentage β = −0.17, SE = 0.076, *p* = 0.026 No covariates were reported
4^X^ Marceau et al. (52); United States (Developed)	Cohort	361 (43%)	4.50–9	White, African, American, Hispanic, Latino, Multiethnic, other	Saliva (morning)	DELFIA	BMI	BMI was calculated based on measured weight and height	Associations between morning salivary cortisol and BMI: β = −1.34, 95% CI: −2.28, −0.4, *p* < 0.05 No covariates were reported
4^z^ Marceau et al. (52); United States (Developed)	Cohort	361 (43%)	4.50–9	White, African, American, Hispanic, Latino, Multiethnic, other	Saliva (evening)	DELFIA	BMI	BMI was calculated based on measured weight and height	Associations between evening salivary cortisol and BMI: β = −0.52, 95% CI: −3.52, 2.48 No covariates were reported
5^1^*Lynch et al. (51); United States (Developed)	Cross-sectional	147 (57.1%)	10–12	African American, Asian, Caucasian, Hispanic	Saliva (mid-morning) (9:30 A.M.–11:00 A.M.)	ELISA	Waist Circumference	Waist circumference was measured	NR
5^2^* Lynch et al. (51); United States (Developed)	Cross-sectional	147 (57.1%)	10–12	African American, Asian, Caucasian, Hispanic	Saliva (mid-morning) (9:30 A.M.–11:00 A.M.)	ELISA	BMI	BMI was calculated based on measured weight and height	NR
6 Lu et al. (34); China (Developing)	Cross-sectional	85 (45.9%)	11.4 0 ± 0.30	Chinese, not otherwise specified	Saliva cortisol (lnAUCi, TSST-C)	LC-MS/MS	BMI z-score	BMI was calculated based on measured weight and height. BMI z-score is defined as the number of standard deviation units from the mean or reference value	Associations between salivary cortisol lnAUCi and BMI: β = 0.051, 95% CI: −1.74, 1.84 No covariates were reported
7^1^ Papafotiou et al. (35); Greece (Developed)	Cross-sectional	50 (100%)	7.60 ± 1.30	Greek	Saliva (AUCg)	ECLIA	BMI z-score	BMI was calculated based on measured height and weight. BMI z-score was generated based on Cole’ s international criteria (Cole, Bellizzi, 2000)	Correlations between salivary cortisol (AUCg) and BMI z-score: *r* = 0.352, *p* = 0.016 No covariates were reported
7^2x^ Papafotiou et al. (35); Greece (Developed)	Cross-sectional	50 (100%)	7.60 ± 1.30	Greek	Saliva (morning)	ECLIA	BMI z-score	BMI was calculated based on measured height and weight. BMI z-score was generated based on Cole’ s international criteria (Cole, Bellizzi, 2000)	Correlations between morning salivary cortisol and BMI-Z score: *r* = 0.321, *p* = 0.031 No covariates were reported
7^3z^ Papafotiou et al. (35); Greece (Developed)	Cross-sectional	50 (100%)	7.60 ± 1.30	Greek	Saliva (evening)	ECLIA	BMI z-score	BMI was calculated based on measured height and weight. BMI z-score was generated based on Cole’ s international criteria (Cole, Bellizzi, 2000)	Correlations between salivary cortisol and BMI z-score: *r* = 0.413, *p* = 0.006 No covariates were reported
8 Chu et al. (49); China (Developing)	Cross-sectional	110 (50.9%)	4–5	Chinese, not otherwise specified	Saliva (morning)	LC-MS/MS	BMI	BMI was calculated based on measured weight and height	Correlations between salivary cortisol and BMI: *r* = 0.001, *p* = 0.426 No covariates were reported
9^1^ Lu et al. (45); China (Developing)	Cross-sectional	87 (44.8%)	12–13	Chinese, not otherwise specified	Saliva (AUCi, after TSST-C)	ELISA	BMI	BMI was based on measured weight and height	Correlations between salivary cortisol (AUCi) and BMI: *r* = 0.15 No covariates were reported
9^2$^ Lu et al. (45); China (Developing)	Cross-sectional	87 (44.8%)	12–13	Chinese, not otherwise specified	Saliva (AUCi, after TSST-C)	ELISA	Body fat percentage	Percentage body fat was measured by BIA-method	Correlations between salivary cortisol (AUCi) and PBF: *r* = 0.15 No covariates were reported
10^x^ Ruttle et al. (23); United States (Developed)	Cohort	323 (NR)	11–18	Largely Caucasian	Saliva (morning)	ELISA	BMI	BMI was calculated based on measured weight and height	Associations between morning salivary cortisol and BMI: β = −0.17, 95% CI: −0.29, −0.05, *p* < 0.01 Covariates included: Child sex and pubertal status, maternal BMI
10^y^ Ruttle et al. (23); United States (Developed)	Cohort	323 (NR)	11–18	Largely Caucasian	Saliva (afternoon)	ELISA	BMI	BMI was calculated based on measured weight and height	Associations between afternoon salivary cortisol and BMI: β = −0.15, 95% CI: −0.27, −0.03, *p* < 0.01 Covariates included: Child sex and pubertal status, maternal BMI
10^z^ Ruttle et al. (23); United States (Developed)	Cohort	323 (NR)	11–18	Largely Caucasian	Saliva (evening)	ELISA	BMI	BMI was calculated based on measured weight and height	Associations between evening salivary cortisol and BMI: β = −0.12, 95% CI: −0.24, −0.002, *p* < 0.01 Covariates included: Child’s sex and pubertal status, maternal BMI
11^1$^ Miller et al. (46); United States (Developed)	Cross-sectional	218 (50.9%)	4.40 ± 0.58	White, Black, Biracial, Hispanic/Latino	Saliva (AUC, Stress-elicitation challenge tasks)	ELISA	BMI z-score	BMI was calculated based on measured weight and height. BMI z-score was calculated based on US Centers for Disease Control reference growth curves for age and sex	Associations between salivary cortisol (AUC) and BMI z-score: β = −0.17, 95% CI: −0.31, −0.03, *p* = 0.018 Covariates included: Child’s: Age, sex, ethnicity Maternal: Overweight and family income-to-needs ratio
11^2$^ Miller et al. (62); United States (Developed)	Cohort	115 (NR)	4.40 ± 0.58	White, Black, Biracial, Hispanic/Latino	Saliva (AUC, Stress-elicitation challenge tasks)	ELISA	Change of BMI z-score	BMI was calculated based on measured weight and height. BMI z-score was calculated based on US Centers for Disease Control reference growth curves for age and sex	Associations between salivary cortisol (AUC) and change of BMI-Z score: β = 0.002, 95% CI: −0.004, 0.008, *p* = 0.410 Covariates included: Child’s: Age, sex, ethnicity Maternal: Overweight and family income-to-needs ratio
12^c^ Francis et al. (31); United States (Developed)	Cross-sectional	32 (NR)	5–7	White, Black, others	Saliva (AUCi, TSST-C)	ELISA	BMI z-score	BMI was extracted from medical record	Associations between salivary cortisol (AUCi) and BMI z-score: β = 0.07, 95% CI: −0.32, 0.46 Covariates included: Child’s: Eating in the absence of hunger Parental: Combined education
12^d^ Francis et al. (31); United States (Developed)	Cross-sectional	11 (NR)	8–9	White, Black, others	Saliva (AUCi, TSST-C)	ELISA	BMI z-score	BMI was extracted from medical record	Associations between salivary cortisol (AUCi) and BMI z-score: β = 1.38, 95% CI: 0.46, 2.30, *p* < 0.01 Covariates included: Child’s: Eating in the absence of hunger Parental: Combined education
13^a$^ Hill et al. (50); United States (Developed)	Cohort	153 (0%)	9.60 ± 0.90	Caucasian, African American	Saliva (morning)	ELISA	Change in BMI z-score	BMI was calculated based on measured weight and height	Correlations between morning salivary cortisol and change in BMI z-score: *r* = 0.15, *p* = 0.009 No covariates were reported
13^b$^ Hill et al. (50); United States (Developed)	Cohort	163 (100%)	9.60 ± 0.90	Caucasian, African American	Saliva (morning)	ELISA	Change in BMI z-score	BMI was calculated based on measured weight and height	Correlations between morning salivary cortisol and change in BMI z-score: *r* = –0.015
14^a^ Dockray et al. (30); United States (Developed)	Cross-sectional	56 (0%)	11.44	Hispanic, Hispanic, African American, Asian American	Saliva cortisol (lnAUCi, TSST-C)	ELISA	BMI	BMI was calculated based on measured weight and height	Correlations between saliva cortisol lnAUCi and BMI: *r* = 0.29, *p* < 0.05 No covariates were reported
14^b^ Dockray et al. (30); United States (Developed)	Cross-sectional	55 (100%)	10.49	Hispanic, Hispanic, African American, Asian American	Saliva cortisol (logAUCi, TSST-C)	ELISA	BMI	BMI was calculated based on measured weight and height	Correlations between saliva cortisol logAUCi and BMI: *r* = 0.52, *p* < 0.01 No covariates were reported
15 Barat et al. (48); France (Developed)	Cross-sectional	19 (63.2%)	6–13	Not reported	Saliva (morning)	RIA	Truncal distribution of fat mass (TDFM)	TDFM was assessed with dual energy X-ray absorptiometry	Correlations between morning salivary cortisol and TDFM: *r* = 0.38 for total children, *r* = −0.33 for boys, *r* = 0.53 for girls No covariates were reported
16 Rosmalen et al. (47); Netherlands (Developed)	Cross-sectional	894 (100%)	10–12	Not reported	Saliva (AUCi, normal condition)	TRFIA	BMI	BMI was calculated based on measured weight and height	Correlation between salivary cortisol (AUCi) and BMI: *r* = 0.072, *p* = 0.042 No covariates were reported
**Serum cortisol**									
1^1^ Gallagher et al. (67); Greek (Developed)	Cross-sectional	2,665 (49.5%)	9–13	Not reported	Serum	ELISA	Visceral fat	Visceral fat was measured by BIA method	Serum cortisol was associated with visceral fat: β = −0.04, 95% CI: −0.1, −0.07, *p* = 0.01 Covariates included: Child’s: Sex, tanner stage, total daily energy intake and total steps per day Maternal: Education
1^2^ Gallagher et al. (67); Greek (Developed)	Cross-sectional	2,665 (49.5%)	9–13	Not reported	Serum	ELISA	BMI	BMI was calculated based on measured weight and height	Serum cortisol was associated with visceral fat: β = −0.03, 95% CI: −0.1, 0.0, *p* = 0.06 Covariates included: Child’s: Sex, tanner stage, total daily energy intake and total steps per day Maternal: Education
1^3^ Gallagher et al. (67); Greek (Developed)	Cross-sectional	2,665 (49.5%)	9–13	Not reported	Serum	ELISA	BMI z-score	BMI z-score was calculated based on WHO 2007 growth reference for age	Serum cortisol was associated with BMI z-score: β = −0.01, 95% CI: −0.0, 0.0, *p* = 0.11) Covariates included: Child’s: Sex, tanner stage, total daily energy intake and total steps per day Maternal: Education
2* Koester-Weber et al. (68); Multi-Centre in Europe (Developed)	Cross-sectional	927 (55%)	14.90 ± 1.20	Not reported	Serum	ELISA	Overweight vs. Non-overweight	BMI was calculated by measuring weight and height. Overweight: BMI > 25 Kg/m^2^, Obesity: BMI > 30 kg/m^2^	NR
3^1$^ Hillman et al. (53); United States (Developed)	Cross-sectional	218 (100%)	14.90 ± 2.20	White, Black, Other	Serum (afternoon and AUCi)	RIA	BMI z-score	BMI was calculated based on measured weight and height. BMI z-score was generated based on the CDC growth charts of U.S (2000)	Associations between serum cortisol (AUCi) and BMI z-score: β = −0.02, 95% CI: −0.04, −0.003, *p* = 0.02 No covariates were reported
3^2$^ Hillman et al. (53); United States (Developed)	Cross-sectional	218 (100%)	14.90 ± 2.20	White, Black, Other	Serum (afternoon and AUCi)	RIA	BMI	BMI was calculated based on measured weight and height	Associations between serum cortisol (AUCi) and BMI: β = −0.06, 95% CI: −0.156, 0.03, *p* = 0.20 Covariates included: Child’s: Age, race, Tanner stage, and socio-economic status
3^3$^ Hillman et al. (53); United States (Developed)	Cross-sectional	218 (100%)	14.90 ± 2.20	White, Black, Other	Serum (afternoon and AUCi)	RIA	Body fat percentage	Percentage body fat was measured by DXA	Associations between serum cortisol (AUCi) and PBF: β = −0.05, 95% CI: −0.17, 0.08, *p* = 0.49 Covariates included: Child’s: Age, race, and socio-economic status
4^1^ Adam et al. (69); United States (Developed)	Cross-sectional	211 (43.6%)	10.80–11.10	Latino	Serum	RIA	BMI	BMI was calculated based on measured weight and height	Correlations between serum cortisol and BMI: *r* = 0.06 No covariates were reported
4^2^ Adam et al. (69); United States (Developed)	Cross-sectional	211 (43.6%)	10.80–11.10	Latino	Serum	RIA	Waist circumference	Waist circumference was measured	Correlations between serum cortisol and WC: *r* = −0.03 No covariates were reported
5 Weigensberg et al. (70); United States (Developed)	Cross-sectional	205 (42.4%)	11.10 ± 1.70	Latino	Serum	RIA	Waist circumference	Waist circumference was measured	Correlations between serum cortisol and WC: *r* = 0.02 No covariates were reported
6 Barat et al. (48); France (Developed)	Cross-sectional	39 (43.6%)	6–13	Not reported	Serum	RIA	Truncal distribution of fat mass (TDFM)	TDFM was assessed with dual energy X-ray absorptiometry	Correlations between morning salivary cortisol and TDFM: *r* = 0.17 for total children, *r* = 0.33 for boys, *r* = 0.40 for girls No covariates were reported
**Urine cortisol**									
1^1$^ Hillman et al. (53); United States (Developed)	Cross-sectional	218 (100%)	14.90 ± 2.20	White, Black, Other	Urine free cortisol (afternoon)	RIA	BMI	BMI was calculated based on measured weight and height. BMI z-score was generated based on the CDC growth charts of U.S (2000)	Associations between urine cortisol and BMI: β = 3.54, 95% CI: 1.12, 5.97, *p* = 0.005 Covariates included: Child’s: Age, race, Tanner stage, and socio-economic status
1^2$^ Hillman et al. (53); United States (Developed)	Cross-sectional	218 (100%)	14.90 ± 2.20	White, Black, Other	Urine free cortisol (afternoon)	RIA	BMI z-score	BMI was calculated based on measured weight and height	Associations between urine cortisol and BMI z-score: β = 0.56, 95% CI: 0.16, 0.96, *p* = 0.007 Covariates included: Child’s: Race, Tanner stage, and socio-economic status
1^3$^ Hillman et al. (53); United States (Developed)	Cross-sectional	218 (100%)	14.90 ± 2.20	White, Black, Other	Urine free cortisol (afternoon)	RIA	Body fat percentage	Percentage body fat was measured by DXA	Associations between urine cortisol and PBF: β = 2.60, 95% CI: −0.65, 5.85, *p* = 0.12 Covariates included: Child’s: Age, race, and socio-economic status
2$ Barat et al. (48); France (Developed)	Cross-sectional	28 (50%)	6–13	Not reported	Urine free cortisol morning	RIA	Truncal distribution of fat mass (TDFM)	TDFM was assessed with dual energy X-ray absorptiometry	Correlation between urine cortisol and TDFM was: *r* = −0.28 for total children, *r* = 0.09 for boys and *r* = 0.25 for girls No covariates were reported

*BMI, body mass index; WC, waist circumference; PBF, percentage body fat; BMI-SDS, BMI standard deviation score; SDS, standard deviation score; FMI, fat mass index; FFMI, free fat mass index; WtHR, waist to height ratio; CDC, Centers for Disease Control and Prevention; CI, confidence interval; M, mean; NR, not reported;β, beta coefficient; ELISA, enzyme-linked immunosorbent assay; CLIA, chemiluminescence immunoassay; HPLC-MS/MS, high-performance liquid chromatography-tandem mass spectrometry; LC-MS/MS, liquid chromatography tandem mass spectrometry; ECLIA, electrochemiluminescence immunoassay; RIA, radioimmunoassay; DELFIA, dissociation-enhanced lanthanide fluorescence immunoassay; TRFIA, a time-resolved fluorescence immunoassay; TSST-C, Trier Social Stress Test for Children; AUC, area under the curve with respect to ground; AUCi, saliva cortisol area-under-the-curve-increase.*

*^#^The two cohort studies that showed associations between hair cortisol and weight status were not included in the meta-analysis because the explanation of effect size (β) is different.*

*^#^These studies were not included in the meta-analysis because the meaning of effect size (β) was different as those of the other studies.*

*These studies were not included in the meta-analysis because the effect sizes were not reported.

*^$^These studies were not included in the meta-analysis because the studies reported the homologous association < 2.*

*a and b: The studies data was extracted from one publication by gender, a for boys and b for girls, respectively.*

*c and d: The studies data was extracted from one publication in the age groups.*

*x, y, and z: Study data were extracted from one publication according to different measurement times – x for morning, y for afternoon and z for evening. In meta-analysis, we only included the association between morning cortisol and weight status.*

### Hair Cortisol Concentration and Adiposity-Related Outcomes Among Children

Nineteen articles encompassing 11,067 children reported on associations between HCC and adiposity-related outcomes, with three longitudinal articles, 11 articles among children aged ≤ 12 years old, 16 from developed countries, 13 using 3 cm hair samples, and ten extracting cortisol by ELISA and eight by LC-MS/MS. All articles measured BMI/BMI z-score/BMI-SDS, and six of them also measured WC, PBF, FMI-SDS/FMI z-score, and WtHR (Table 1).

**FIGURE 2:**
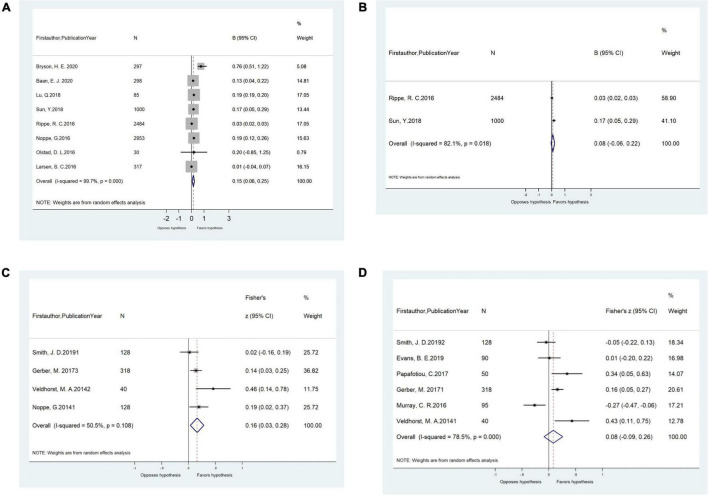
**(A)** Meta-analysis of the adjusted associations (β, 95% CI) between hair cortisol and BMI/BMI z-score in cross-sectional studies (*n* = 8). **(B)** Meta-analysis of the adjusted associations (β, 95% CI) between hair cortisol and FMI-SDS/FMI z-score in cross-sectional studies (*n* = 2). **(C)** Meta-analysis of the unadjusted correlations (*r*, 95% CI) between hair cortisol and waist circumference in cross-sectional studies (*n* = 4). **(D)** Meta-analysis of the unadjusted correlations (*r*, 95% CI) between hair cortisol and BMI/BMI z-score/BMI-SDS in cross-sectional studies (*n* = 6). BMI, body mass index; BMI SDS, BMI standard deviation scores.

Unadjusted correlations (*r*) between HCC and WC were significant (*n* = 4 studies, pooled-*r* = 0.16, 95% CI: 0.03, 0.28; Figure 2C). Similar unadjusted correlations were found for studies extracting HCC by ELISA (*n* = 3 studies, pooled-*r* = 0.19, 95% CI: 0.03, 0.40) and for studies by CLIA (*n* = 1 study, *r* = 0.14, 95% CI: 0.03, 0.25). However, the unadjusted correlations between HCC and BMI/BMI z-score/BMI-SDS were not significant (Figure 2D). Significant unadjusted correlations between HCC and BMI/BMI z-score were found for girls (*n* = 2 studies, pooled-*r* = 0.20, 95% CI: 0.07, 0.34) but not for boys (*n* = 1 study, *r* = 0.13, 95% CI: −0.03, 0.29; Table 2).

**TABLE 2:** Overall and sub-group meta-analysis of the associations between hair cortisol concentration and adiposity-related outcome(s) among children based on 20 included studies.

					Heterogeneity				
Sample	Adiposity-related outcome(s)	N of studies	Effect size (β, 95% CI)	*P*-value	I^2^ (%)	χ^2^	*P*-value	Tau-squared	*P*-value (Egger’s test)*^e^*
(1) Cross-sectional studies (β, 95% CI)									
Overall	FMI-SDS/FMI z-score	2	0.08 (−0.06, 0.22)	024	82.1	5.60	0.02	0.01	–
Overall*^a^*	BMI/BMI z-score	8	**0.15 (0.06, 0.25)**	0.002	99.7	2,200.16	<0.001	0.01	0.69
Age group									
≤12 years	BMI/BMI z-score	6	**0.15 (0.05, 0.26)**	<0.001	99.8	2,199.92	<0.001	0.01	
>12 years	BMI/BMI z-score	2	**0.13 (0.04, 0.22)**	0.004	0	0.02	0.90	<0.001	
Country developmental context									
Developed countries	BMI/BMI z-score	6	**0.12 (0.03, 0.21)**	<0.001	88.4	43.22	<0.001	0.01	
Developing countries	BMI/BMI z-score	2	**0.193 (0.188, 0.198)**	<0.001	0	0.14	0.71	<0.001	
Measurement method									
LC-MS/MS	BMI/BMI z-score	3	**0.18 (0.06, 0.29)**	0.002	99.8	2186.67	<0.001	0.01	
ELISA	BMI/BMI z-score	5	0.08 (−0.06, 0.22)	0.26	65.1	5.74	0.06	0.01	
(2) Cross-sectional studies (*r*, 95% CI)									
Overall^a,c^	Waist circumference	4	**0.16 (0.03, 0.28)**	0.01	50.5	6.07	0.11	0.01	0.449
Measurement method									
ELISA	Waist circumference	3	**0.19 (0.03, 0.40)**	0.01	67.0	6.06	0.05	0.02	
CLIA^d^	Waist circumference	1	**0.14 (0.03, 0.25)**	–	–	–	–	–	
Overall^c^	BMI/BMI z-score/BMI-SDS	6	0.08 (−0.09, 0.26)	0.35	78.5	23.29	<0.001	0.04	0.918
Measurement method									
ELISA	BMI/BMI z-score/BMI-SDS	3	0.02 (−0.32, 0.35)	0.93	84.5	12.88	0.002	0.07	
LC-MS/MS	BMI/BMI z-score/BMI-SDS	2	0.16 (−0.16, 0.48)	0.33	69.8	3.31	0.07	0.04	
Sex^b^									
Boys^d^	BMI/BMI z-score	1	0.13 (−0.03, 0.29)	–	–	–	–	–	
Girls	BMI/BMI z-score	2	**0.21 (0.06, 0.36)**	0.003	13.9	1.16	0.28	<0.001	

*FMI, fat mass index; BMI, body mass index; SDS, standard deviation score; ELISA, Enzyme-Linked Immunosorbent Assay; LC–MS/MS, liquid chromatography tandem mass spectrometry; CLIA, chemiluminescence immunoassay.*

*We had searched two cohort studies that reported the associations (β, 95% CI) between hair cortisol concentration and adiposity, one of the studies showed that the associations between hair cortisol concentration and BMI: β (95% CI) = 4.62 (1.41, 7.83) (p < 0.01), the other one’s effective size has different meaning. Thus, we were unable to perform a meta-analysis.*

*^a^These studies did not report the associations between cortisol and adiposity for boys and girls, respectively. Thus, we did not do the subgroups analysis across genders, country context, or age groups.*

*^b^Among the six studies showed association (r) between hair cortisol concentration and weight status, only one reported the association for boys and girls. We divided the article into two studies and one study only showed the association for girls.*

*^c^These studies were all from developed countries and the participants were ≤12 years old, thus, we did not perform sub-group meta-analysis across country context and age groups.*

*^d^In the sub-group meta-analysis, only one study was included and the effect size was the one reported in the original study.*

*^e^The Egger’s tests was used to indicate the existence of publication bias. If p-value < 0, it was indicated that publication bias was existed, otherwise, no publication bias existed. Numbers in bold indicate significance.*

In meta-analyses, the pooled adjusted associations from cross-sectional studies revealed that HCC was positively associated with FMI-SDS/FMI z-score (*n* = 2 studies, pooled-β = 0.04, 95% confidence interval [CI]: 0.01, 0.08) and BMI/BMI z-score (*n* = 8 studies, pooled-β = 0.15, 95% CI: 0.06, 0.25; Figures 2A,B). Such adjusted associations varied by cortisol measurement method. Significant effects were found for studies extracting HCC by LC-MS/MS (*n* = 3 studies, pooled-β = 0.18, 95% CI: 0.06, 0.29) but not for those by ELISA (*n* = 5 studies, pooled-β = 0.08, 95% CI: −0.06, 0.22). Similar adjusted associations were observed for children aged ≤ 12 years old (*n* = 6 studies, pooled-β = 0.15, 95% CI: 0.05, 0.26) and children > 12 years old (*n* = 2 studies, pooled-β = 0.13, 95% CI: 0.04, 0.22), and for studies from developing countries (*n* = 2 studies, pooled-β = 0.193, 95% CI: 0.188, 0.198) and those from developed countries (*n* = 6 studies, pooled-β = 0.12, 95% CI: 0.03, 0.21; Table 2).

### Salivary Cortisol Concentration and Adiposity-Related Outcomes Among Children

Sixteen articles with 3,462 children examined associations between salivary cortisol concentration and adiposity-related outcomes, including 13 cross-sectional articles and five longitudinal articles (two articles reported both cross-sectional and longitudinal results). Fourteen of the 16 articles examined children ≤ 12 years old, twelve articles took place in developed countries, five articles examined cortisol as AUCi (area-under-the-curve-increase) and two reported AUCg (area under the curve with respect to ground), and eleven articles used ELISA for cortisol extraction. All these articles measured BMI/BMI z-score and four also measured WC and PBF (Table 1).

**FIGURE 3:**
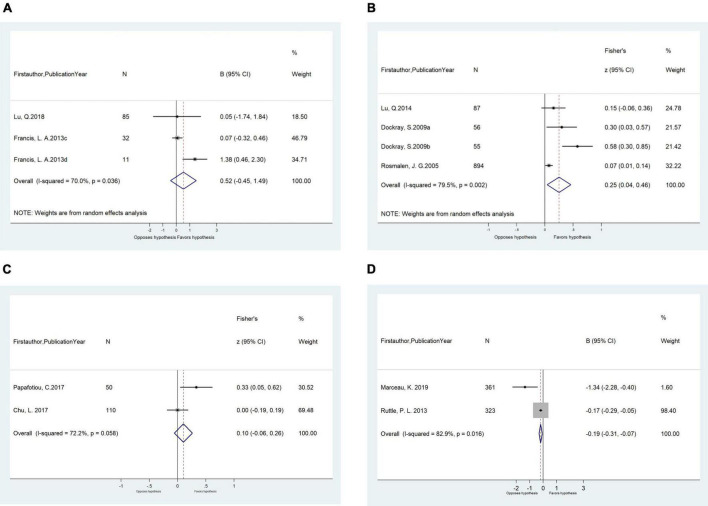
**(A)** Meta-analysis of the associations (β, 95% CI) between salivary cortisol (lnAUCi) and BMI z-score in cross-sectional studies (*n* = 3). **(B)** Meta-analysis of the unadjusted correlations (*r*, 95% CI) between salivary cortisol (log AUCi) and BMI in cross-sectional studies (*n* = 4). **(C)** Meta-analysis of the unadjusted correlations (*r*, 95% CI) between morning salivary cortisol and BMI/BMI z-score in cross-sectional studies (*n* = 2). **(D)** Meta-analysis of the longitudinal adjusted effects (β, 95% CI) of morning salivary cortisol on BMI in longitudinal studies (*n* = 2). BMI, body mass index.

In meta-analyses, the total daily cortisol output of salivary cortisol (as AUCi) was positively correlated with BMI among all children (*n* = 4 studies, pooled-*r* = 0.25, 95% CI: 0.04, 0.46) in cross-sectional studies (Figure 3B). Age and country developmental context modified such unadjusted correlations. Significant correlations were found for studies among children aged ≤ 12 years old (*n* = 3 studies, pooled-*r* = 0.30, 95% CI: 0.02, 0.61) but not for children > 12 years old (*n* = 1 study, *r* = 0.15, 95% CI: −0.06, 0.37), and for studies from developed countries (*n* = 3 studies, pooled-*r* = 0.30, 95% CI: 0.02, 0.61) but not for the study from developing country (*n* = 1 study, *r* = 0.15, 95% CI: −0.06, 0.37). The significant pooled correlations were similar for studies extracting salivary cortisol using ELISA (*n* = 3 studies, pooled-*r* = 0.33, 95% CI: 0.09, 0.58) and using TRFIA (*n* = 1 study, *r* = 0.07, 95% CI: 0.01, 0.14), and for study among boys (*n* = 1 study, *r* = 0.30, 95% CI: 0.03, 0.57) and girls (*n* = 2 studies, pooled-*r* = 0.10, 95% CI: 0.04, 0.16; Table 3).

**TABLE 3:** Overall and sub-group meta-analysis of the associations between salivary cortisol concentration and adiposity-related outcomes among children based on 11 included studies.

					Heterogeneity				
Sample	Adiposity outcomes	N of studies	Effect size (β, 95% CI)	*P* value	I^2^ (%)	χ^2^	*P*-value	Tau-squared	*P*-value (Egger’s test)^c^
1. Total daily output of salivary cortisol (lnAUCi or logAUCi, cross-sectional studies)									
(1) Salivary cortisol (lnAUCi; β, 95% CI)^a^	BMI z-score	3	0.52 (−0.45, 1.49)	0.29	70.0	6.66	0.04	0.48	0.655
Measurement method									
LC-MS/MS^b^	BMI z-score	1	0.05 (−1.74, 1.84)	0.96	–	–	–	–	
ELISA	BMI z-score	2	0.66 (−0.62, 1.93)	0.31	84.9	6.60	0.01	0.73	
Country developmental context									
Developing country^b^	BMI z-score	1	0.05 (−1.74, 1.84)	0.96	–	–	–	–	
Developed country	BMI z-score	2	0.66 (−0.62, 1.93)	0.31	84.9	6.60	0.01	0.73	
(2) Salivary cortisol (logAUCi; *r*, 95% CI)	BMI	4	**0.25 (0.04, 0.46)**	0.02	79.5	14.6	0.002	0.03	0.147
Measurement method									
ELISA	BMI	3	**0.33 (0.09, 0.58)**	<0.001	65.6	5.82	0.06	0.03	
TRFIA^b^	BMI	1	**0.07 (0.01, 0.14)**	0.03	–	–	–	–	
Sex									
Boys^b^	BMI	1	**0.30 (0.03, 0.57)**	0.03	–	–	–	–	
Girls	BMI	2	0.31 (−0.19, 0.80)	0.002	92.0	12.49	<0.001	0.12	
Country developmental context									
Developed countries	BMI	3	**0.30 (0.02,0.61)**	<0.001	86.2	14.47	0.001	0.06	
Developing countries^b^	BMI	1	0.15 (−0.06, 0.37)	<0.001	–	–	–	–	
Age group	BMI								
≤12 years	BMI	3	**0.30 (0.02, 0.61)**	<0.001	86.2	14.47	0.001	0.07	
>12 years	BMI	1	0.15 (−0.06, 0.37)	0.17	–	–	–	–	
(3) Morning salivary cortisol (*r*, 95% CI, cross-sectional studies)	BMI/BMI z-score	2	0.15 (−0.17, 0.47)	0.367	72.2	3.59	0.06	0.04	–
(4) Morning salivary cortisol (β, 95% CI, cohort studies	BMI	2	−**0.66 (**−**1.79, 0.47)**	0.25	82.9	5.86	0.02	0.57	–

*BMI, body mass index; AUCi, saliva cortisol area-under-the-curve-increase; LC-MS/MS, liquid chromatography tandem mass spectrometry; ELISA, enzyme-linked immunosorbent assay; TRFIA, a time-resolved fluorescence immunoassay.*

*^a^Age of the children in the three studies were ≤12 years, and they did not report the association between salivary cortisol concentration and BMI/BMI z-score for boys or girls. Thus, we did not do the subgroups analysis across gender and age groups.*

*^b^In the sub-group meta-analysis, only one study was included, so the data reported in the original study was presented.*

*^c^The Egger’s tests was used to indicate the existence of publication bias. If p-value < 0, it was indicated that publication bias was existed, otherwise, no publication bias existed. Numbers in bold indicate significance.*

However, the adjusted association between salivary cortisol concentration (as AUCi) and BMI z-score was non-significant (*n* = 3 studies, pooled-β = 0.52, 95% CI: −0.45, 1.49; Figure 3A). The associations were also non-significant stratifying by cortisol measurement method (LC-MS/MS vs. ELISA) and country developmental context (developing country vs. developed country; Table 3).

Regarding morning salivary cortisol, neither its correlations with BMI/BMI z-score from two cross-sectional studies (pooled-*r* = 0.10 95% CI: *r* = −0.06, 0.26) nor the adjusted associations from two cohort studies were significant (pooled-β = −0.19, 95% CI: −0.31, −0.07; Table 3 and Figures 3C,D).

### Serum Cortisol Concentration and Adiposity-Related Outcomes Among Children

**FIGURE 4:**
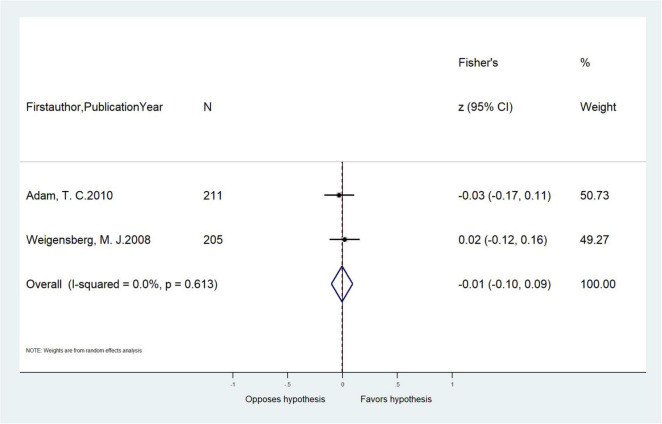
Meta-analysis of the unadjusted correlations (*r*, 95% CI) between serum cortisol concentration and waist circumference in cross-sectional studies (*n* = 2).

Six cross-sectional articles encompassing 4,265 children examined associations between serum cortisol concentration and adiposity-related outcomes. All were based in developed countries. Three articles were among children aged ≤ 12 years old and four articles extracted cortisol by RIA. Two articles measured BMI/BMI z-score, while others measured WC, PBF, visceral fat, and TDFM (Table 1). Pooled results showed that serum cortisol concentration was not correlated with WC (pooled-*r* = −0.01, 95% CI: −0.10, 0.09) from two cross-sectional studies (Table 4 and Figure 4). Meta-analysis of serum cortisol concentration and other adiposity-related outcomes were not possible due to insufficient statistical data.

**TABLE 4:** Overall meta-analysis of the correlations (*r*, 95% CI) between serum cortisol concentration and waist circumference among children based on cross-sectional studies.

					Heterogeneity				
Sample	Adiposity outcome(s)	N of studies	Correlations (*r*, 95% CI)	*P*-value	I^2^ (%)	χ^2^	*P*-value	Tau-squared	*P*-value Egger’s test)^a^
Overall	Waist circumference	2	−0.01 (−0.10, 0.09)	0.91	0	0.26	0.61	< *0*.001	–

*^a^The Egger’s tests was used to indicate the existence of publication bias. If p-value < 0, it was indicated that publication bias was existed, otherwise, no publication bias existed.*

### Urinary Cortisol Concentration and Adiposity-Related Outcomes Among Children

Two articles of 246 children examined associations between urinary cortisol and adiposity-related outcomes. Both were from developed countries and extracted cortisol by RIA. One study measured TDFM while the other measured BMI/BMI z-score and PBF (Table 1). Of the two articles, one reported that the correlations between urinary cortisol and TDFM was *r* = 0.14 (−0.24, 0.49) for all children and by sex, for boys: *r* = 0.09, and for girls: *r* = 0.25. The other article reported that urinary cortisol to be positively associated with BMI (β = 3.54, 95% CI: 1.12, 5.97) and BMI z-score (β = 0.56, 95% CI: 0.16, 0.96), but not with PBF (β = 2.60, 95% CI: −0.65, 5.85). Further subgroup meta-analysis was not possible because necessary statistics were not available.

### Sensitivity Analysis and Assessment of Publication Bias

Respective sensitivity analyses were conducted to examine associations of HCC, salivary cortisol, and serum cortisol concentration with adiposity-related outcomes. Only when the study by Chu et al., 2017 was removed from the meta-analyses of cross-sectional studies did the non-significant correlations between morning salivary cortisol concentration and BMI/BMI z-score become significant (*r* = 0.35, 95% CI: 0.10, 0.60; Supplementary Table 3). The Egger’s tests and funnel plots indicated no publication bias within our evaluated study parameters (Table 2 and Supplementary Figure 1).

## Discussion

This is the first systematic review and meta-analysis to synthesize and evaluate the associations between different cortisol measures and adiposity-related outcomes in children. We found that most of our included studies examined the associations of either HCC or salivary cortisol concentration with adiposity-related outcomes, and most studies were from developed countries. However, results from our meta-analysis indicated that only HCC, the cortisol measure that serves as an indicator of long-term stress and cumulative cortisol activity, was positively associated with objectively measured adiposity-related outcomes (i.e., FMI-SDS/FMI z-score, BMI, BMI z-score) in children. Salivary, serum, and urinary cortisol measures were not consistently associated with these adiposity-related outcomes, especially after adjustment for covariates, and/or lacked sufficient data for meta-analyses.

For HCC, meta-analysis of results from cross-sectional studies showed it to be robustly and positively associated with objectively measured adiposity-related outcomes in children, including FMI-SDS/FMI z-score and BMI/BMI z-score. The age- (≤12 years vs. >12 years) and country developmental context-stratified (developing countries vs. developed countries) analyses also supported these positive adjusted associations. Our meta-analyses result also revealed HCC to be positively correlated with WC without adjusting for covariates. These observations support the role of chronic stress or chronically elevated levels of cortisol in the development and maintenance of both general and central obesity in children. These findings are consistent with the results of a previous systematic review (21). Cortisol increases fat accumulation *via* glucocorticoid receptors, which have a particularly high density in visceral adipose tissue (38). Moreover, cortisol can increase food intake, especially of energy dense “comfort foods”(39), which can further contribute to increased obesity risk. The positive pooled effect sizes between HCC and adiposity-related outcomes corroborate the importance of considering chronic stress exposures over more acute stress measures when designing or evaluating childhood obesity interventions as well as in treating obesity (7).

Notably, our meta-analyses revealed the novel importance of HCC measurement method, the choice of which modified adjusted cross-sectional associations between HCC and BMI/BMI z-score in children. Only HCC extracted by LC-MS/MS, not ELISA, was associated with BMI/BMI z-scores. Immunoassays such as ELISA tend to yield higher but less accurate HCC than LC-MS/MS, possibly because ELISA overestimate steroid content given antibody cross-reactivity (40). Rather, LC-MS/MS offers superior specificity and sensitivity with its multi-analyte capabilities, making it the preferred method for HCC analysis in high-quality clinical research (41). Additionally, thanks to the high sensitivity for cortisol in hair provided by mass spectrometers, only small samples of hair are needed to run LC-MS/MS, which is conducive for large epidemiological studies among pediatric populations. Future studies should measure HCC by LC-MS/MS, and more longitudinal work is necessary to examine long-term associations.

Twelve of the 17 studies measuring HCC used hair 3 cm proximal to the scalp. Based on an average hair growth rate of 1 cm per month, such samples can reflect the cumulative cortisol and cortisone secretion of HPA axis in the previous 3 months (42). It follows then that most studies using HCC are, either consciously or not, accounting for chronic stress over the past 3-months in children. Other studies have also suggested that researchers could retrospectively examine cortisol production for a particular preceding time period when stress could have been more salient (43). However, other studies have observed HCC to decrease gradually along the length of hair shaft as distal hair samples may suffer more insults (e.g., repeated water and soap exposure) (44). Future study designs should consider these attributes and explore ways to incorporate HCC measures so as to capture cortisol levels encompassing several months. This will serve to further elucidate associations between chronic stress and childhood obesity.

In contrast to the long-term inference enabled by HCC, salivary cortisol concentration is more reflective of HPA reactivity and the stress response facilitated by laboratory settings (30). Seven (30, 31, 34, 35, 45–47) of the 13 studies (23, 30, 31, 34, 35, 45–52) used AUC_*i*_ to assess increases in salivary cortisol after administering the Trier Social Stress Test for Children (TSST-C) (53). Though AUC_*i*_ of salivary cortisol was correlated with BMI prior to adjusting for covariates, the adjusted associations were not significant for cross-sectional or longitudinal studies, for studies that measured salivary cortisol by ELISA or LC-MS/MS or for studies from developing or developed countries.

Rather than AUCi of salivary cortisol, the other six studies (23, 48–52) measured morning salivary cortisol to indicate the cortisol awakening response (54). However, we found neither unadjusted nor adjusted associations between morning salivary cortisol concentration and adiposity-related outcomes to be significant. These findings suggest that both cortisol awakening response and cortisol reactivity to acute stress challenge tasks are not associated with adiposity-related outcomes in children. Correspondingly, recent longitudinal studies found that obesity predicted greater changes in cortisol awakening response and cortisol reactivity to challenge in early to middle childhood, not that cortisol awakening response and cortisol reactivity predicted increased likelihood of obesity over the same time period (31). In our review, only four of the 13 included studies were longitudinal, precluding similar inferences on the direction of these associations. More longitudinal studies are needed to understand these associations.

Given the current evidence base, serum cortisol concentration was not observed to be correlated with WC and BMI in children. For urinary cortisol and adiposity-related outcomes, limited studies and data precluded further meta-analyses. However, we did have two studies examine these associations, both supporting significant positive associations between urinary cortisol and BMI (34). Still, these studies’ cross-sectional designs and solitary existence demonstrate the need for more efforts to confirm serum and urinary cortisol associations in childhood obesity.

The present systematic review and meta-analysis expands the knowledge base concerning stress biomarker utility in pediatric adiposity research by providing pooled effect sizes for different cortisol measures against objectively measured adiposity-related outcomes. These findings may help health professionals and policymakers better understand how different cortisol measures reflect underlying stress processes and how stress may contribute to adiposity in children. This review also comprehensively investigated the effects of potential moderators on cortisol-adiposity associations, such as age, sex, cortisol measurement method, and country development context. These latter findings provide insights on how to measure HCC more precisely, and how to better understand obesogenic effects of stress in different socio-demographic and economic contexts. Furthermore, examining the pooled effect sizes separately using unadjusted and adjusted models provides a more comprehensive picture of the cortisol with adiposity.

Nonetheless, some limitations should be considered in the interpretation of our results. First, sex-stratified analyses of adjusted associations between HCC and adiposity-related outcomes were not possible given limited statistics available. Second, the generalizability of our findings is limited as we included only studies published in English, most of our included studies were from developed countries, and we excluded studies focusing on children with mental disorders or chronic diseases. Third, most studies were observational in nature, precluding causal interpretations. Fourth, while our findings provide insights on physiological stress processes and adiposity-related outcomes, the sources of stress could not be identified beyond chronicity and acuteness and are thus unable to inform actionable recommendations for childhood obesity prevention efforts; such can be the efforts of future work. Fifth, the number of studies included in some subgroup analyses were small as only limited eligible studies were available, especially for salivary and serum cortisol; more studies utilizing these biomarkers are needed. Last, as several original studies with <50 participants were included in the meta-analysis, the small samples reduced the power to find significant associations between cortisol and adiposity-related outcomes.

After consideration of the four cortisol measures of hair, saliva, serum, and urine in children, this study provides important evidence supporting a positive relationship between HCC and objectively measured adiposity-related outcomes. Similar findings were found for children aged ≤12 years and >12 years, and for children from developing and developed countries. These findings provide direct evidence of the physiological stress processes that contribute to increased risk of adiposity-related outcomes in children, and corroborate the need to focus on chronic stress in childhood obesity intervention efforts.

## Data Availability Statement

The original contributions presented in this study are included in the article/Supplementary Material, further inquiries can be directed to the corresponding authors.

## Author Contributions

LuM and LeM designed the research. XL, LuM, NY, and MC conducted the literature search, data screening, and extraction. XL performed the meta-analysis. LuM, XL, and DTC drafted the manuscript. LeM and DTC provided administrative support for the project and had primary responsibility for the final manuscript. All authors read and approved the final manuscript. All authors revised the manuscript, critically helped in the interpretation of results, provided relevant intellectual input, and approved the submitted version.

## Conflict of Interest

The authors declare that the research was conducted in the absence of any commercial or financial relationships that could be construed as a potential conflict of interest.

## Publisher’s Note

All claims expressed in this article are solely those of the authors and do not necessarily represent those of their affiliated organizations, or those of the publisher, the editors and the reviewers. Any product that may be evaluated in this article, or claim that may be made by its manufacturer, is not guaranteed or endorsed by the publisher.
