# Supplementary material for: Associations Between Different Cortisol Measures and Adiposity in Children: A Systematic Review and Meta-Analysis
Source: Front Nutr. 2022 Jun 23;9:879256. doi: 10.3389/fnut.2022.879256 (PMC9260431; doi:10.3389/fnut.2022.879256)
Supplement: Supplementary file 1 [file Data_Sheet_1.docx]

Supplementary Material

**Associations between different cortisol status measures and adiposity in children: A systematic review and meta-analysis**

**Supplemental Table 1. Search strategies of the associations between different cortisol status measures and adiposity among children**

| Cortisol measures | #1 Title/Abstract: cortisol, HPA*, cortisone | #1 AND #2  AND #3 |
| --- | --- | --- |
| Adiposity-related outcomes | #2 Title/Abstract: obes*, overweight*, adiposity*, fat*, heavy, heaviness, weight, excess weight, body mass index, central fat or obesity, skin fat, waist circumference, ration of waist to hip. |  |
| Population | #3 Title/Abstract: teenager*, adolescen*, child*, youth, preadult, teen*, juvenile*, pubescent*, pubert*, pediatric*, school age*, young*, school child*, kid* |  |

**Supplemental Table 2. Quality assessment of the included studies using the National Heart, Lung, and Blood Institute’s Quality Assessment Tool for Observational Cohort and Cross-Sectional Studies.**

| Study ID / First author,  Publication year | 1 | 2 | 3 | 4 | 5 | 6 | 7 | 8 | 9 | 10 | 11 | 12 | 13 | 14 | Total ^a^ | Quality |
| --- | --- | --- | --- | --- | --- | --- | --- | --- | --- | --- | --- | --- | --- | --- | --- | --- |
| Hair1/ Petimar, J.  2020 | Yes | Yes | Yes | Yes | No | Yes | Yes | Yes | Yes | Yes | Yes | NA | NR | Yes | 11 | High |
| Hair2/ Bryson, H. E.  2020 | Yes | Yes | Yes | Yes | No | No | No | Yes | Yes | Yes | Yes | NA | NA | Yes | 9 | High |
| Hair3/ Baan, E. J.  2020 | Yes | Yes | NR | Yes | No | No | No | Yes | Yes | Yes | Yes | NA | NA | NR | 7 | High |
| Hair4/ Smith, J. D.  2019 | Yes | Yes | Yes | Yes | No | No | No | Yes | Yes | Yes | Yes | NA | NA | Yes | 9 | High |
| Hair5/ Evans, B. E.  2019 | Yes | Yes | Yes | Yes | No | Yes | Yes | Yes | Yes | Yes | Yes | NA | NA | Yes | 9 | High |
| Hair6/ Distel, L. M. L.  2019 | Yes | Yes | Yes | Yes | No | Yes | Yes | Yes | Yes | Yes | Yes | NA | NR | Yes | 11 | High |
| Hair7/ Sun, Y.  2018 | Yes | Yes | NR | Yes | No | No | No | Yes | Yes | Yes | Yes | NA | NA | Yes | 8 | High |
| Hair8salivary3/ Lu, Q. Y.  2018 | Yes | Yes | Yes | Yes | No | No | No | Yes | Yes | Yes | Yes | NA | NA | NR | 8 | High |
| Hair9salivary4/ Papafotiou, C.  2017 | Yes | Yes | NR | Yes | No | No | No | Yes | Yes | Yes | Yes | NA | NA | NR | 7 | High |
| Hair10/ Gerber, M.  2017 | Yes | Yes | No | Yes | No | No | No | Yes | Yes | Yes | Yes | NA | NA | NR | 7 | High |
| Hair11/ Rippe, R. C.  2016 | Yes | Yes | Yes | Yes | No | No | No | Yes | Yes | Yes | Yes | NA | NA | Yes | 9 | High |
| Hair12/ Olstad, D. L.  2016 | Yes | Yes | No | Yes | No | No | No | Yes | Yes | Yes | Yes | NA | NA | Yes | 8 | High |
| Hair13/ Noppe, G.  2016 | Yes | Yes | Yes | Yes | No | No | No | Yes | Yes | Yes | Yes | NA | NA | Yes | 9 | High |
| Hair14/ Murray, C. R.  2016 | Yes | Yes | Yes | Yes | No | No | No | Yes | Yes | Yes | Yes | NA | NA | Yes | 9 | High |
| Hair15/ Larsen, S. C.  2016 | Yes | Yes | Yes | Yes | No | No | No | Yes | Yes | Yes | Yes | NA | NA | Yes | 9 | High |
| Hair16/ Veldhorst, M. A.  2014 | Yes | Yes | NR | Yes | No | No | No | Yes | Yes | Yes | Yes | NA | NA | NR | 7 | High |
| Hair17/ Noppe, G.  2014 | Yes | Yes | NR | Yes | No | No | No | Yes | Yes | Yes | Yes | NA | NA | Yes | 8 | High |
| Salivary1/ Marceau, K.  2019 | Yes | Yes | Yes | Yes | No | Yes | Yes | Yes | Yes | Yes | Yes | NA | No | NR | 10 | High |
| Salivary2/ Lynch, T.  2019 | Yes | Yes | NR | Yes | No | No | No | Yes | Yes | Yes | Yes | NA | NA | NR | 7 | High |
| Salivary5/ Chu, L.  2017 | Yes | Yes | NR | Yes | No | No | No | Yes | Yes | Yes | Yes | NA | NA | Yes | 8 | High |
| Salivary6/ Lu, Q. Y.  2014 | Yes | Yes | Yes | Yes | No | No | No | Yes | Yes | Yes | Yes | NA | NA | NR | 8 | High |
| Salivary7/ Ruttle, P. L.  2013 | Yes | Yes | Yes | Yes | No | Yes | Yes | Yes | Yes | Yes | Yes | NA | N0 | NR | 10 | High |
| Salivary8/ Miller, A. L.  2013 | Yes | Yes | Yes | Yes | No | Yes | Yes | Yes | Yes | Yes | Yes | NA | NR | Yes | 11 | High |
| Salivary9/ Francis, L. A.  2013 | Yes | Yes | NR | Yes | No | No | No | Yes | Yes | Yes | Yes | NA | NA | NR | 7 | High |
| Salivary10/ Hill, E. E.  2011 | Yes | Yes | Yes | Yes | No | Yes | Yes | Yes | Yes | Yes | Yes | NA | NR | Yes | 11 | High |
| Salivary11/ Dockray, S.  2009 | Yes | Yes | No | Yes | No | No | No | Yes | Yes | Yes | Yes | NA | NA | Yes | 8 | High |
| Salivary12 serum5 urine2/ Barat, P.  2007 | Yes | Yes | No | Yes | No | No | No | Yes | Yes | Yes | Yes | NA | NA | Yes | 8 | High |
| Salivary13/ Rosmalen, J. G. M.  2005 | Yes | Yes | NR | Yes | No | No | No | Yes | Yes | Yes | Yes | NA | NA | NR | 7 | High |
| Serum1/ Koester-Weber,T  2014 | Yes | Yes | NR | Yes | Yes | No | No | Yes | Yes | Yes | Yes | NA | NA | NR | 8 | High |
| Serum2urine1/ Hillman, J. B.  2012 | Yes | Yes | NR | Yes | No | No | No | Yes | Yes | Yes | Yes | NA | NA | Yes | 8 | High |
| Serum3/ Adam, T. C.  2010 | Yes | Yes | NR | Yes | No | No | No | Yes | Yes | Yes | Yes | NA | NA | Yes | 8 | High |
| Serum4/ Weigensberg, M. J.  2008 | Yes | Yes | No | Yes | No | No | No | Yes | Yes | Yes | Yes | NA | NA | Yes | 8 | High |

Note: The 14 items are as follows:

1. Was the research question or objective in this paper clearly stated?

2. Was the study population clearly specified and defined?

3. Was the participation rate of eligible persons at least 50%?

4. Were all the subjects selected or recruited from the same or similar populations (including the same time period)? Were inclusion and exclusion criteria for being in the study prespecified and applied uniformly to all participants?

5. Was a sample size justification, power description, or variance and effect estimates provided?

6. For the analyses in this paper, were the exposure(s) of interest measured prior to the outcome(s) being measured?

7. Was the timeframe sufficient so that one could reasonably expect to see an association between exposure and outcome if it existed?

8. For exposures that can vary in amount or level, did the study examine different levels of the exposure as related to the outcome?

9. Were the exposure measures clearly defined, valid, reliable, and implemented consistently across all study participants?

10. Was the exposure(s) assessed more than once over time?

11. Were the outcome measures clearly defined, valid, reliable, and implemented consistently across all study participants?

12. Were the outcome assessors blinded to the exposure status of participants?

13. Was loss to follow-up after baseline 20% or less?

14. Were key potential confounding variables measured and adjusted statistically for their impact on the relationship between exposure(s) and outcome(s)?

a: Overall quality was rated based on the total score of the scale, with 0-3, 4-7, and 7-14 reflecting poor, fair, and good quality, respectively.

**Supplemental Table 3. Sensitivity analysis**

1. Meta-analysis of the associations (β, 95%CI) between hair cortisol concentration and BMI/BMI z-score in cross-sectional studies (n=8)

| Study omitted | Effect Size (95%CI) | I^2^ (%) | χ^2^ | P-value |
| --- | --- | --- | --- | --- |
| Bryson, H. E. 2020 | 0.12 (0.02,0.22) | 99.7 | 2187.24 | <0.001 |
| Baan, E. J. 2020 | 0.15 (0.05,0.26) | 99.7 | 2199.95 | <0.001 |
| Sun, Y. 2018 | 0.15 (0.05,0.25) | 99.7 | 2199.17 | <0.001 |
| Lu, Q. 2018 | 0.13 (0.05,0.21) | 87.7 | 48.73 | <0.001 |
| Rippe, R. C. 2016 | 0.17 (0.08,0.25) | 88.9 | 53.95 | <0.001 |
| Olstad, D. L. 2016 | 0.15 (0.05,0.25) | 99.7 | 2200.13 | <0.001 |
| Noppe, G. 2016 | 0.14 (0.04,0.24) | 99.7 | 2195.00 | <0.001 |
| Larsen, S. C. 2016 | 0.18 (0.07,0.28) | 99.7 | 2187.68 | <0.001 |

1. Meta-analysis of the associations (r, 95%CI) between hair cortisol concentration and waist circumference in cross-sectional studies (n=4)

| Study omitted | Effect Size (95%CI) | I^2^ (%) | χ^2^ | P-value |
| --- | --- | --- | --- | --- |
| Smith, J. D. 2019 | 0.20 (0.07,0.34) | 41.1 | 3.40 | 0.18 |
| Gerber, M. 2017 | 0.19 (-0.03,0.40) | 67.0 | 6.06 | 0.05 |
| Veldhorst, M. A. 2014 | 0.12 (0.04,0.21) | 7.4 | 2.16 | 0.34 |
| Noppe, G. 2014 | 0.16 (-0.02,0.34) | 65.0 | 5.71 | 0.06 |

1. Meta-analysis of the unadjusted correlations (r, 95%CI) between hair cortisol concentration and BMI/BMI z-score/BMI-SDS in cross-sectional studies (n=6)

| Study omitted | Effect Size (95%CI) | I^2^ (%) | χ^2^ | P-value |
| --- | --- | --- | --- | --- |
| Smith, J. D. 2019 | 0.11 (-0.10,0.33) | 80.9 | 20.99 | <0.001 |
| Evans, B. E. 2019 | 0.10 (-0.11,0.31) | 82.5 | 22.86 | <0.001 |
| Papafotiou, C. 2017 | 0.41 (-0.15,0.23) | 79.8 | 19.79 | 0.001 |
| Gerber, M. 2017 | 0.07 (-0.15,0.29) | 79.1 | 19.11 | 0.001 |
| Murray, C. R. 2016 | 0.15 (-0.003,0.29) | 63.3 | 10.91 | 0.03 |
| Veldhorst, M. A. 2014 | 0.03 (-0.15,0.21) | 78.2 | 18.33 | 0.03 |

1. Meta-analysis of the associations (β, 95%CI) between salivary cortisol (ln AUCi) and BMI z-score in cross-sectional studies (n=3)

| Study omitted | Effect Size (95%CI) | I^2^ (%) | χ^2^ | P-value |
| --- | --- | --- | --- | --- |
| Lu, Q. 2018 | 0.66 (-0.62,1.93) | 84.9 | 6.60 | 0.01 |
| Francis, L. A. 2013 | 0.95 (-0.28,0.17) | 40.4 | 1.68 | 0.20 |
| Francis, L. A. 2013 | 0.07 (-0.31,0.45) | 0 | 0 | 0.98 |

1. Meta-analysis of the unadjusted correlations (r, 95%CI) between salivary cortisol (log AUCi) and BMI in cross-sectional studies (n=4)

| Study omitted | Effect Size (95%CI) | I^2^ (%) | χ^2^ | P-value |
| --- | --- | --- | --- | --- |
| Lu, Q. 2014 | 0.30 (-0.02,0.61) | 86.2 | 14.47 | 0.001 |
| Dockray, S. 2009a | 0.24 (-0.02,0.50) | 84.2 | 12.69 | 0.002 |
| Dockray, S. 2009b | 0.12 (0.01,0.23) | 31.1 | 2.90 | 0.23 |
| Rosmalen, J.G. 2005 | 0.33 (0.08,0.58) | 65.6 | 5.82 | 0.06 |

**Supplemental Figure 1. Funnel plot for publication bias**

a) Cross-sectional studies examining associations between hair cortisol concentration and BMI/BMI z-score (β)


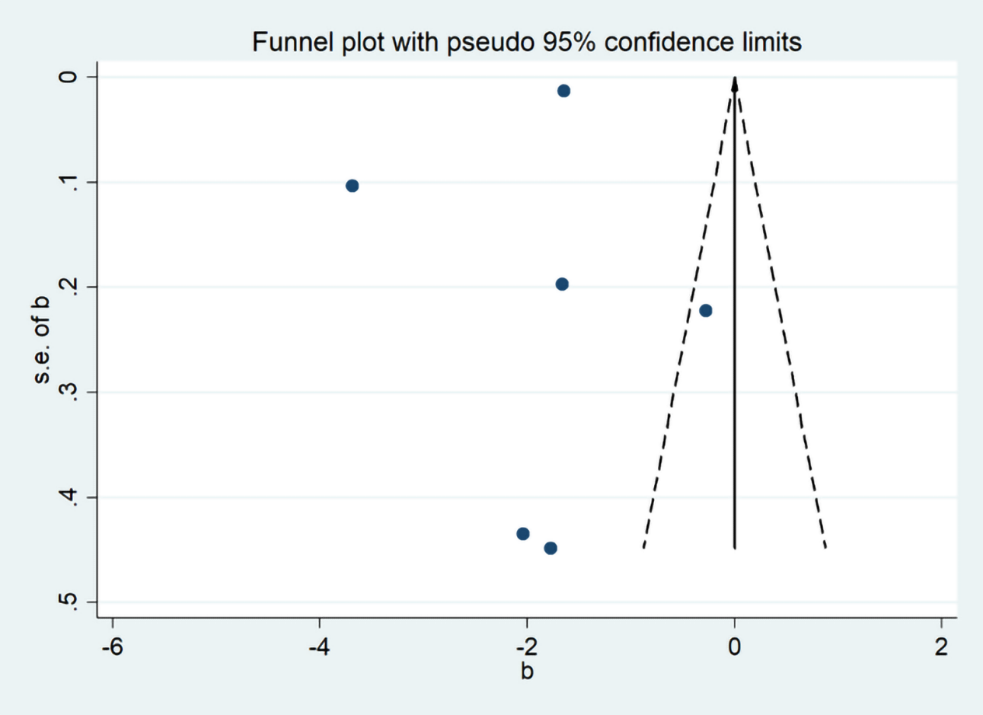


b) Cross-sectional studies examining correlations between hair cortisol concentration and BMI/BMI-SDS/BMI z-score (r)


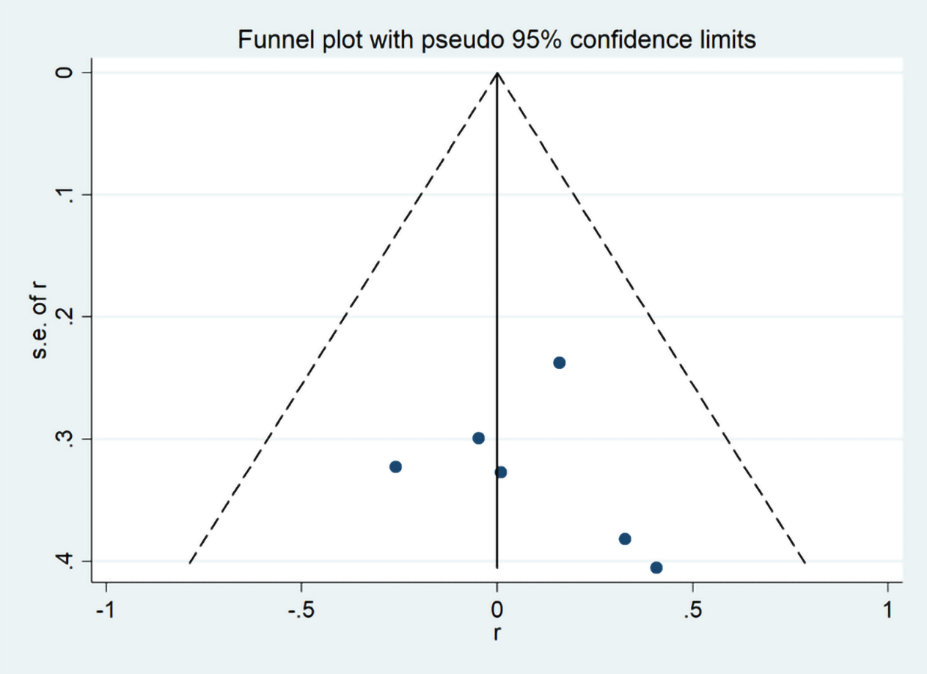


c) Cross-sectional studies examining correlations between hair cortisol concentration and waist circumference (r)


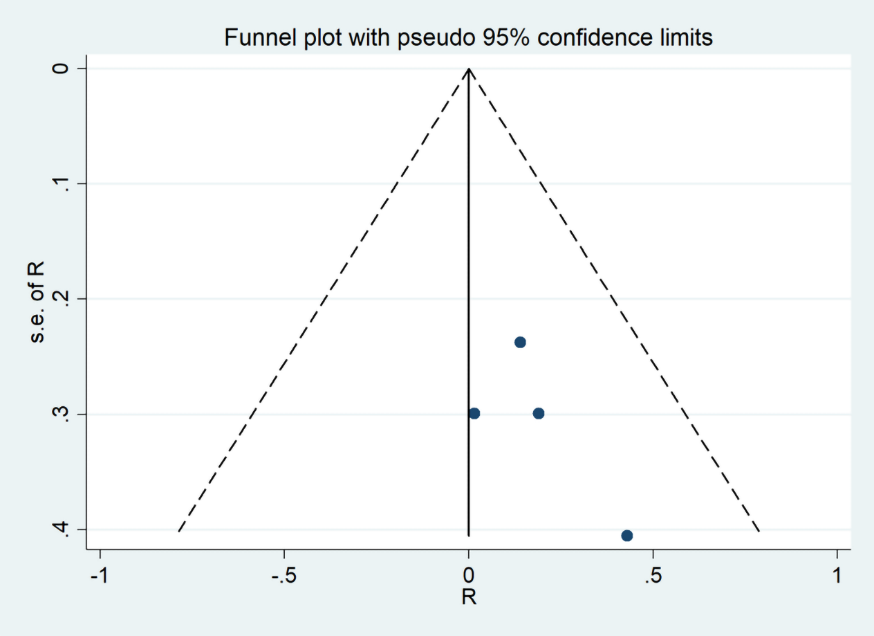


d) Cross-sectional studies examining associations between salivary cortisol (lnAUCi) and BMI z-score (β)


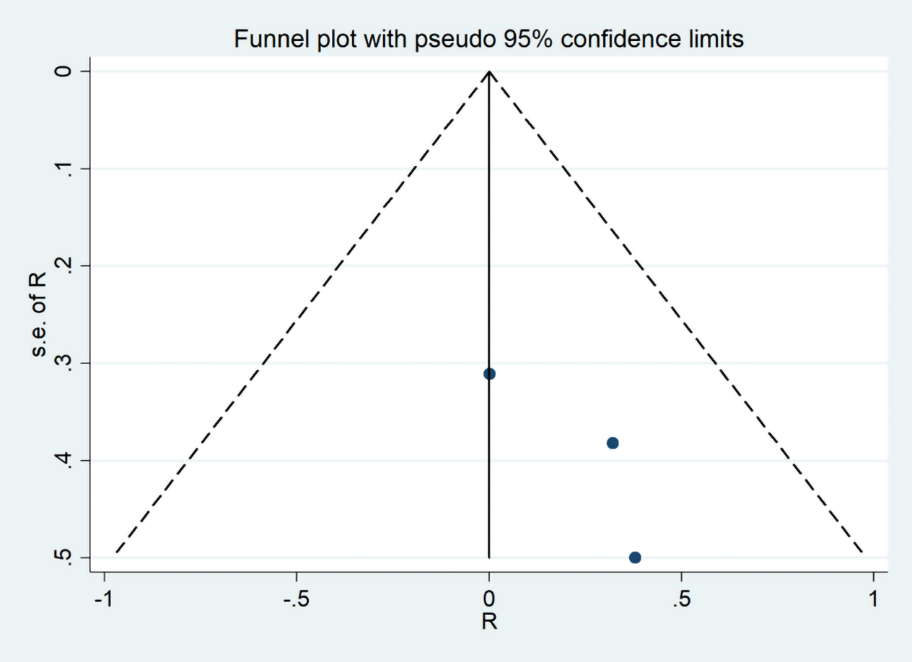


Non-AUCi

e) Cross-sectional studies examining correlations between salivary cortisol (logAUCi) and BMI (r)


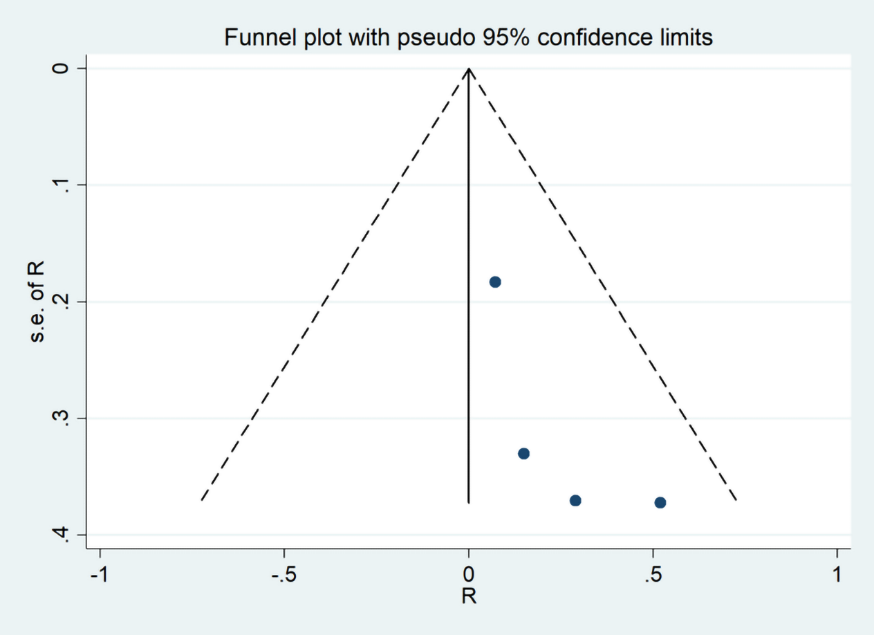


AUCi

Abbreviations: BMI: body mass index
